# Network Pharmacologic Analysis of *Dendrobium officinale* Extract Inhibiting the Proliferation of Gastric Cancer Cells

**DOI:** 10.3389/fphar.2022.832134

**Published:** 2022-03-25

**Authors:** Lianping Wei, Wenhao Dong, Zhen Han, Chen Chen, Qing Jin, Jinling He, Yongping Cai

**Affiliations:** ^1^ School of Life Science, Anhui Agricultural University, Hefei, China; ^2^ Mabwell (Shanghai) Bioscience Co., Ltd., Shanghai, China

**Keywords:** *D. officinale*, gastric cancer, network pharmacology, protein-protein interaction, MTT

## Abstract

Globally, gastric cancer (GC) is one of the three most deadly cancers. *Dendrobium officinale* (*D. officinale*) is a traditional Chinese medicine (TCM), and its extract can significantly inhibit the proliferation of gastric cancer cells. However, there are no unified conclusions on its potential active components and possible mechanisms of action. This paper aims at exploring the potential active components, targets, and cell pathways of *D. officinale* extract in inhibiting the proliferation of gastric cancer cells by using network pharmacology and cytology experiments. In this paper, UPLC-MS/MS was used to identify the main chemical components in the extracts of *D. officinale*, and the an ADME model was used to screen the potential active components. Network pharmacology methods such as target prediction, pathway identification, and network construction were used to determine the mechanism through which the *D. officinale* extract inhibited gastric cancer cell proliferation. MTT assays, fluorescence confocal microscopy, clone formation, and flow cytometry were used to verify the inhibitory activity of the *D. officinale* extract on gastric cancer cell proliferation *in vitro*. The UPLC-MS/MS analysis identified 178 chemical components from the *D. officinale* extract. Network pharmacology analysis showed that 13 chemical components had the potential to inhibit the proliferation of gastric cancer cells, with the possible involvement of 119 targets and 20 potential signaling pathways. *In vitro* experiments confirmed that the *D. officinale* extract could significantly inhibit the proliferation of gastric cancer cells. Therefore, we believe that the *D. officinale* extract can inhibit the proliferation of gastric cancer cells through effects on multiple components, multiple targets, and multiple pathways.

## Introduction

Gastric cancer is a malignant tumor with high morbidity and mortality rates (Joshi and Badgwell, 2021). Although the incidence of gastric cancer has decreased in recent years, it is still the third leading cause of cancer-related death in the world. The 5-year relative survival rate of patients with gastric cancer in most countries is still less than 30% (Chen et al., 2016). Global cancer statistics for 2018 indicate more than 1 million new cases of gastric cancer and an estimated 783,000 deaths (Bray et al., 2018). Although surgical resection and chemotherapy are used to improve the therapeutic effect of gastric cancer, their prognosis is not optimistic. Therefore, the development of new anti-gastric cancer drugs will be of great significance.

In contrast to the approach of Western medicine, Chinese medicine researchers generally believe that the multiple compounds in a Chinese herbal medicine can act on multiple various cell targets through different means, which achieves the therapeutic effects with fewer side effects and less drug resistance. According to preliminary studies, Chinese herbal medicine treatments can be helpful to maintain a reduced tumor size and alleviate tumor-related symptoms (Ling et al., 2014). In addition, studies have shown that Chinese herbal medicine alone or in combination with conventional chemotherapy or radiotherapy can improve the immunity and quality of life of patients with tumors (Liu et al., 2017; Xiao and Tao, 2017; Yang et al., 2019). However, the presence of multiple compounds in Chinese herbal medicine also leads to the complexity of its molecular mechanism. Consequently, revealing the mechanism of the multiple components of Chinese herbal medicines is of great significance in promoting the clinical application of Chinese herbal medicine in the field of antitumor research.

*D. officinale* is a perennial herb belonging to the genus Dendrobium of the family Orchidaceae. As a traditional Chinese herbal medicine and health food, it has been used in China for thousands of years and offers great value for medicine development value and excellent application prospects. Existing studies have shown that *D. officinale* contains an abundance of diverse secondary metabolites, including polysaccharides, alkaloids, stilbenes (phenanthrenes, bibenzyls), sesquiterpenes, and flavonoids; these compounds, have broad-spectrum biological effects, enhance the immune function of the body (Li et al., 2020), and exert antitumor and adjuvant antitumor effects (Zhang et al., 2020). They are commonly used in the clinical adjuvant treatment of malignant tumors and provide a potential resources for the research and development of antitumor drugs.

**FIGURE 1:**
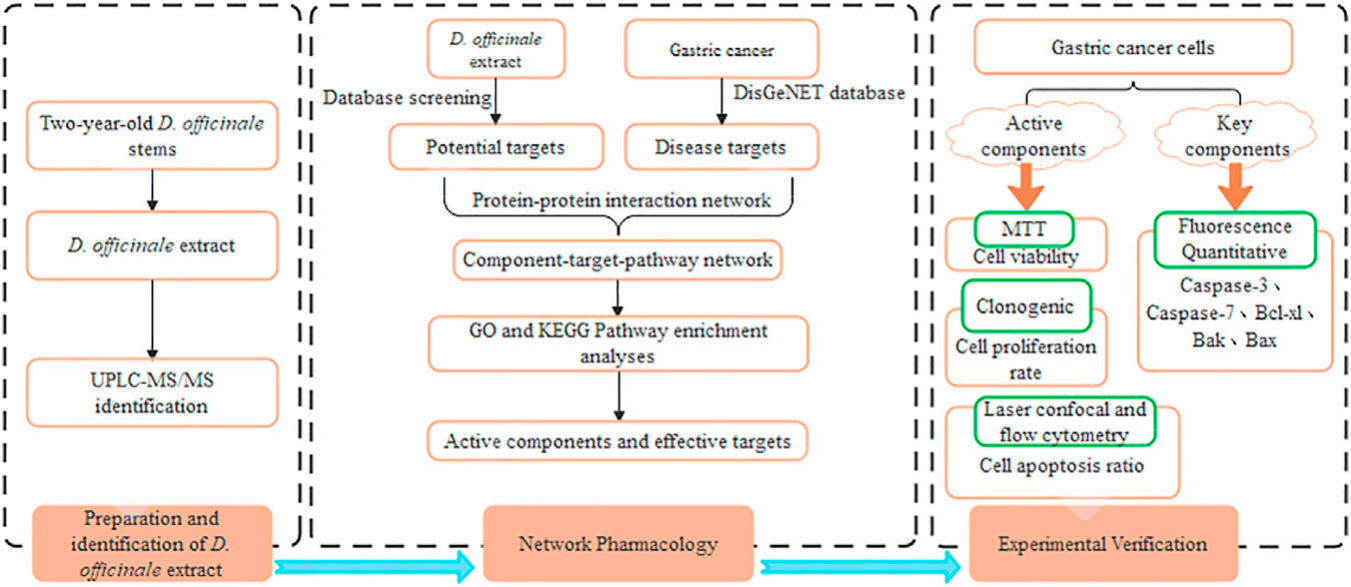
Flowchart of the network pharmacological and experimental studies of the *D. officinale* extract in human gastric cancer cells.

In this study, we identified the main bioactive substances of the *D. officinale* extract. Through a series of network pharmacological analyses including target prediction and enrichment, pathway analysis, and network construction were carried out to determine the potential mechanism of GC-related targets and the *D. officinale* extract. In addition, to confirm the inhibitory effect of the *D. officinale* extract on the proliferation of gastric cancer cells, a series of cytological experiments including cell proliferation, monoclonal formation, and apoptosis were performed in three gastric cancer cell lines SGC-7901, MGC-803, and MKN-45. A schematic diagram of the network pharmacological analysis and overall experimental flow for the *D. officinale* extract in this study is shown in Figure 1. The results of our study not only proved the synergistic anticancer properties of the compounds in *D. officinale* extract but also provided further insight into the molecular mechanism through which the *D. officinale* extract inhibits the proliferation of GC cell.

## Methods

### Preparation of the *Dendrobium officinale* extract

*D. officinale* 2-year fresh stems were taken, placed at 105°C for 30 min, dried in a 60°C oven, and ground into powder, through a 40-mesh sieve. An appropriate amount of *D. officinale* powder was weighed, added with anhydrous ethanol at the ratio of 1:20, soaked at room temperature for 48 h, and ultrasonically extracted at 55°C for 45 min (120 W, 60 HZ). The obtained extract was centrifuged at 6,000 r/min for 10 min. The supernatant was taken and filtered with a .22-μm nylon membrane, then blown to constant weight with nitrogen. An appropriate amount of pure water was added to the above extract to form a suspension, then extracted with an equal volume of petroleum ether, the lower phase was collected, an equal volume of n-butanol was added to continue extraction, and the lower phase was collected and blown to constant weight with nitrogen, which is the *D. officinale* extract.

### Ultra-Performance Liquid Chromatography-Tandem Mass Spectrometry

The 20-mg *D. officinale* extract was mixed with 300 μl pre-cooled methanol–water solution (v/v = 7:1), ultrasonically extracted for 30 min, centrifuged at 14,000 r/min for 10 min, and filtered by a .22-μm filter membrane. The samples were analyzed by UPLC-MS/MS (Thermo Finnigan, San Jose, CA, USA). The specific chromatographic conditions were as follows: column temperature was 40°C, flow rate was 0.35 ml/min, and sample volume was 5 μl.

Under positive ion mode, the chromatographic column was ACQUITY™ BEHC8 (100 mm × 2.1 mm, 1.7 μm). Mobile phase A was .1% formic acid–water, and mobile phase B was .1% formic acid–acetonitrile. The capillary temperature is 350°C, and the standard pressure of sheath and auxiliary gas is 45 and 10 MPa, respectively.

Under negative ion mode, the chromatographic column was ACQUITY™ HSS T3 (100 mm × 2.1 mm, 1.8 μm), the mobile phase A was 0.1% formic acid–water, and the mobile phase B was .1% formic acid–acetonitrile. The capillary temperature is 360°C, and the standard pressure of sheath and auxiliary gas is 50 and 13 MPa, respectively.

In the positive and negative ion modes, the liquid chromatography-double partial pressure linear trap-electrostatic field orbital trap tandem mass spectrometer (Thermo Finnigan, San Jose, CA, USA) was used for analysis (scanning range m/z: 50–1,000), and the spray voltage was +3.5 kV/−3.0 kV.

### Network Pharmacology-Based Analysis

#### Identification of Associated MolecularTargets of *Dendrobium officinale* Extract

The potential molecular targets of the *D. officinale* extract were predicted using the Traditional Chinese Medicines for Systems Pharmacology Database and Analysis Platform (TCMSP) (Huang et al., 2020), the SwissADME (Ge et al., 2019), and the Comparative Toxicogenomics Database (CTD) (Davis et al., 2021).

The GC-associated human genes were comprehensively retrieved from DisGeNET (Pinero et al., 2020).

#### Protein-Protein Interaction Network

The online software STRING was used to obtain PPI data (Ge et al., 2018). The online software generates a score for each protein interaction information. The higher the score, the greater the credibility of the interaction between the target proteins. Therefore, in the parameter setting, we have selected the human species, and the confidence value is set to high confidence (.700).

#### Gene Ontology and KEGG Pathway Enrichment Analyses

The GO functional annotation and KEGG pathway analysis were performed on the target proteins in the PPI network using the Metascape database (*p* ≤ .01), so as to explain the role of these target proteins in molecular function, cell localization, biological processes, and signaling pathways involved.

#### Network Construction and Analysis

The network was constructed using Cytoscape 3.8.2 software. The constructed component, target, and signaling pathway network was constructed based on the active components obtained from the analysis, the predicted target proteins, and signaling pathways related to human gastric cancer. The nodes in the network diagram represent the chemical components analyzed or the predicted target proteins and signaling pathways, and the edges represent the compound–target or target–pathway interactions.

### Experimental Verification

#### Chemicals and Reagents

*D. officinale* (2-year-old) was purchased from the Dendrobium base in Huoshan County, Anhui Province, China (simulated field growing environment). All chemical reagents and solutions were of analytical grade or LC-MS mass spectrometry grade, and methanol, acetonitrile, formic acid, and ammonium bicarbonate were purchased from CNW (Dusseldorf, Germany). Fetal bovine serum (FBS) was purchased from Sangon Biotech (Shanghai, China). RPMI Medium Modified 1640 medium and phosphate-buffered saline (PBS) were purchased from HyClone, Thermo Scientific (MA). Thiazolyl Blue Tetrazolium Blue (MTT) was purchased from Sigma (St. Louis, MO, USA). Annexin V-FITC/PI Double Staining Kit was purchased from BD (Franklin Lakes, NJ, USA). RNeasy™ Animal RNA Isolation Kit with Spin Column was purchased from Beyotime (Shanghai, China). *TransStart*® Green qPCR SuperMix and *TransScript*® Reverse Transcriptase [M-MLV,R-NaseH-] were purchased from TransGen Biotech (Beijing, China).

#### Cell Lines and Cell Culture

The Human GC cell lines SGC-7901, MGC-803, and MKN-45 were donated by Dr. Guodong Shen’s research group of the First Affiliated Hospital of University of Science and Technology of China. The cells were placed in RPMI-1640 medium containing 10% FBS and cultured in a carbon dioxide incubator (CO_2_ concentration of 5%, culture temperature of 37°C).

#### MTT Assay

The cell viability was determined by MTT assay. Cells were seeded in 96-well plates and treated with PBS and different concentrations of *D. officinale* extract (.0625, .125, .25,.5, 1, and 2 mg/ml). After incubation in the carbon dioxide incubator for 24 h, 20 μl MTT (5 mg/ml) was added to each well, and the culture was continued for 4 h. Then, MTT was discarded and 150 μl DMSO was added per well. The absorbance was measured at 495 nm by Molecular Devices (San Jose, CA, USA).

#### Clonogenic Assay

The cells were seeded onto 6-well plates (10^4^ cells/well). SGC-7901 cells were treated with 0, .015, .031, .062, .125, and .250 mg/ml *D. officinale* extract for 24 h, respectively. The medium was replaced by fresh 1,640 medium without the *D. officinale* extract and then cultured for 2 weeks. The plate was washed with a PBS buffer solution, and Giemsa staining was used to observe cell colony formation.

#### Laser Confocal Assay

A total of 1 ml of cells growing in the logarithmic phase with a concentration of 10^4^ cells/l was placed in a laser confocal culture dish. After the cells adhered to the wall and grew, the supernatant was discarded. The cells were treated with RPMI 1640 medium containing different concentrations of the *D. officinale* extract, and the same volume of RPMI 1640 medium was used as the control. After 24 h of extraction, the cells were rinsed twice with precooled PBS. Then, 100 μl 1× binding buffer was added, and 5 μl Annexin V-FITC and 5 μl PI were added. The cells were gently mixed and incubated in the dark at room temperature for 15 min, washed with precooled PBS three times, and observed by laser confocal microscopy.

#### Flow Cytometry

The cells in the logarithmic growth phase were placed in a disposable sterile plate, and the supernatant was discarded after adherent growth. The RPMI 1640 medium containing different concentrations of *D. officinale* extract was added, and the RPMI 1640 medium containing equal volumes was used as control. Each treatment group was set up in three parallel groups. After 24 h, the cells were digested with .25% trypsin and washed twice with precooled PBS. After passing through a 200-mesh sieve, 100 μl 1× binding buffer was added, and then 5 μl Annexin V-FITC and 5 μl PI were added. The cells were gently mixed and incubated in the dark at room temperature for 15 min.

#### Fluorescence Quantitative Detection

The cells in the logarithmic growth phase were taken, and the group of control groups without drug treatment was set. The experimental group was added with 0.5 mg/ml *D. officinale* extract. After treatment for 30 min and 2 and 4 h, the relative expression levels of each gene were detected.

## Results

### Identification of Bioactive Components in *Dendrobium officinale* Extract

**FIGURE 2:**
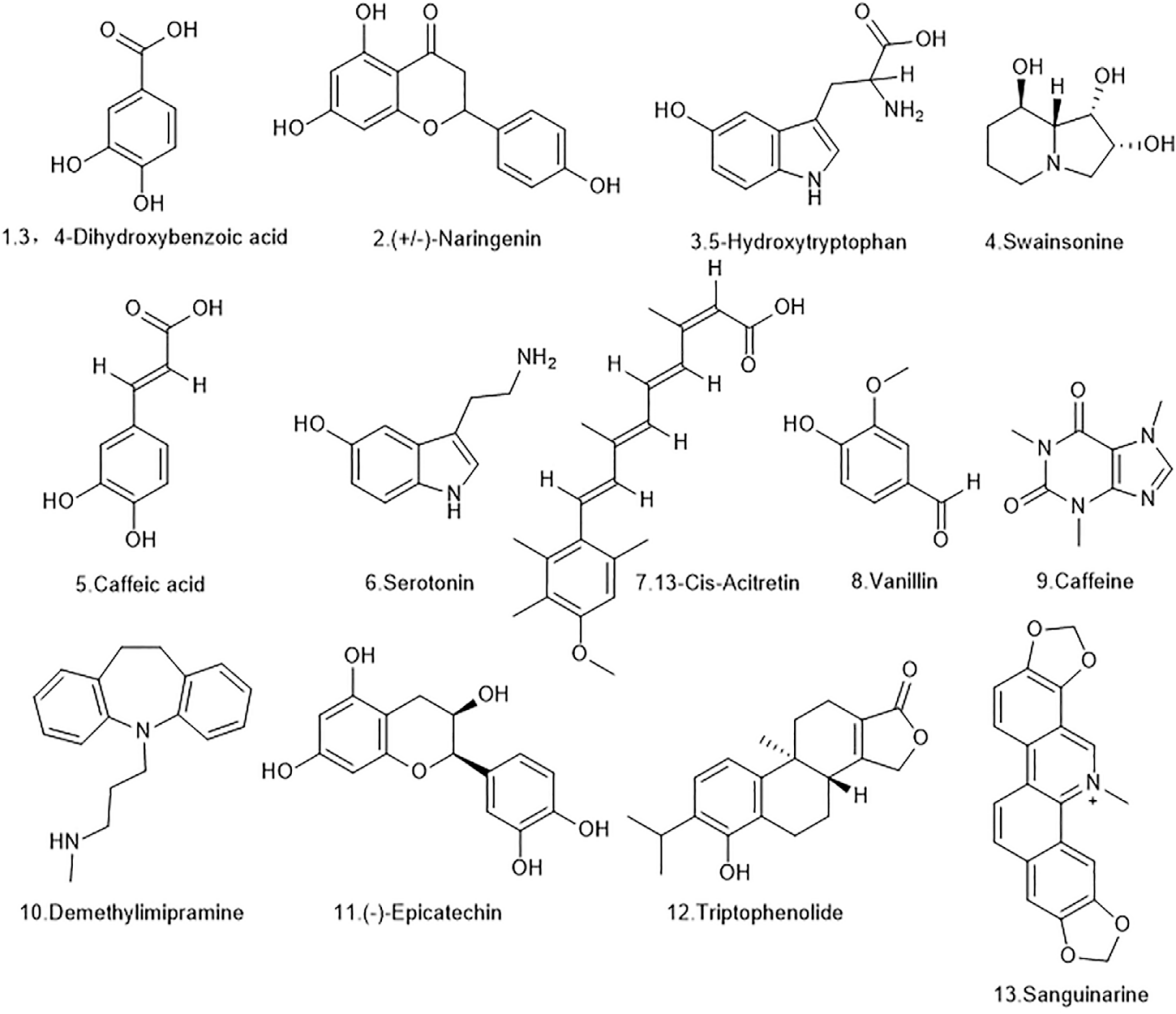
The molecular formulas of the 13 bioactive components identified from *D. officinale* extract.

The extracts of *D. officinale* were determined by UHPLC-LTQ Orbitrap: Ultimate 3000 Velos Pro (Thermo Finnigan, San Jose, CA, USA) and OSI/SMMS (Dalian Dashuo, Dalian, China), and 178 compounds were identified using MS2 and RT/MS1. The 178 identified chemical components include secondary metabolites such as alkaloids, terpenes, and stilbene. The obtained chemical components were analyzed using the TCMSP database and the SwissADME web tool, and the ADME model was used for evaluation. ADME stands for “absorption (A), distribution (D), metabolism (M), and efflux (E),” and represents the processes that drugs are subjected to after entering the body. The screening rules for the TCMSP were OB ≥ 30% and DL ≥ .18. The screening rules for SwissADME were high gastrointestinal absorption (GI absorption) properties in the Pharmacokinetics column and at least two YESs for the first five conditions in the Druglikeness column. Through this screening, 13 qualifying compounds were obtained. The details are shown in Figure 2 and Table 1.

**TABLE 1:** Chemical formulae of the 13 bioactive components identified from *D. officinale* extract.

No	Compound	Chemical formula	m/z
1	3,4-Dihydroxybenzoic acid	C_7_H_6_O_4_	153.0218
2	(±)-Naringenin	C_15_H_12_O_5_	135.0296
3	5-Hydroxytryptophan	C_11_H_12_N_2_O_3_	221.0851
4	Swainsonine	C_8_H_15_NO_3_	174.1103
5	Caffeic acid	C_9_H_8_O_4_	179.0350
6	Serotonin	C_10_H_12_N_2_O	177.1004
7	13-Cis-Acitretin	C_21_H_26_O_3_	325.1838
8	Vanillin	C_8_H_8_O_3_	151.0401
9	Caffeine	C_8_H_10_N_4_O_2_	91.0394
10	Demethylimipramine	C_18_H_22_N_2_	231.1661
11	(-)-Epicatechin	C_15_H_14_O_6_	313.0723
12	Triptophenolide	C_20_H_24_O_3_	311.1678
13	Sanguinarine	C_20_H_14_NO_4_^+^	355.0848

### Identification of GC-Related Targets of *Dendrobium officinale* Extract

Based on the 13 active substances screened, 119 targets were obtained in the Swiss Target Prediction and TCMSP databases (Supplementay Table S1), and 343 genes related to human gastric cancer were identified from the DisGeNET database (Supplementay Table S2). 94 overlapping genes between *D. officinale* extract-associated genes and human gastric cancer-associated genes were obtained by the online tool Venny 2.1.0 software (Table 2).

**TABLE 2:** Gastric cancer-related targets of *D. officinale* extract.

Number	Protein name	Gene name
01	Brain-derived neurotrophic factor	BDNF
02	Cyclin-dependent kinase inhibitor 1	CDKN1A
03	Alpha-enolase	ENO1
04	Interleukin-1 beta	IL1B
05	Interleukin-6	IL6
06	Prostaglandin G/H synthase 2	PTGS2
07	Superoxide dismutase [Mn]	SOD2
08	Tumor necrosis factor	TNF
09	Alpha-fetoprotein	AFP
10	Aryl hydrocarbon receptor	AHR
11	Aldo-keto reductase family 1 member C3	AKR1C3
12	Albumin	ALB
13	Bcl-2-like protein 1	BCL2L1
14	G1/S-specific cyclin-D1	CCND1
15	Interleukin-8	CXCL8
16	Epidermal growth factor receptor	EGFR
17	Heme oxygenase 1	HMOX1
18	Mitogen-activated protein kinase 1	MAPK1
19	Mitogen-activated protein kinase 3	MAPK3
20	Mitogen-activated protein kinase 8	MAPK8
21	Peroxisome proliferator-activated receptor gamma	PPARG
22	Protein kinase C beta type	PRKCB
23	Plasminogen activator inhibitor 1	SERPINE1
24	Zinc finger protein SNAI1	SNAI1
25	Signal transducer and activator of transcription 3	STAT3
26	Cellular tumor antigen p53	TP53
27	Caspase-8	CASP8
28	Polyunsaturated fatty acid 5-lipoxygenase	ALOX5
29	V-type proton ATPase subunit d 2	ATP6V0D2
30	Growth arrest and DNA damage-inducible protein GADD45 alpha	GADD45A
31	Glutathione S-transferase P	GSTP1
32	Nitric oxide synthase	NOS3
33	5′-Nucleotidase	NT5E
34	Peroxiredoxin-5	PRDX5
35	5-Hydroxytryptamine receptor 1A	HTR1A
36	Transcription factor AP-1	JUN
37	40S ribosomal protein S6	RPS6
38	Carbonic anhydrase 1	CA1
39	Carbonic anhydrase 2	CA2
40	Cytochrome P450 2A6	CYP2A6
41	DNA (cytosine-5)-methyltransferase 3B	DNMT3B
42	Fibroblast growth factor receptor 2	FGFR2
43	Metallothionein-2	MT2A
44	Angiotensin-converting enzyme	ACE
45	Type-2 angiotensin II receptor	AGTR2
46	DNA-(apurinic or apyrimidinic site) endonuclease	APEX1
47	Apolipoprotein A-I	APOA1
48	Serine-protein kinase ATM	ATM
49	BH3-interacting domain death agonist	BID
50	BCL2/adenovirus E1B 19 kDa protein-interacting protein 3	BNIP3
51	Caveolin-1	CAV1
52	Cadherin-1	CDH1
53	Cyclin-dependent kinase 4	CDK4
54	Serine/threonine-protein kinase Chk2	CHEK2
55	Procathepsin L	CTSL
56	DNA (cytosine-5)-methyltransferase 1	DNMT1
57	Extracellular matrix protein 1	ECM1
58	Elongation factor 1-alpha 1	EEF1A1
59	Tyrosine-protein kinase Fyn	FYN
60	Gastrin	GAST
61	GTPase HRas	HRAS
62	Heat shock protein beta-1	HSPB1
63	Matrilysin	MMP7
64	Adenine DNA glycosylase	MUTYH
65	Myc proto-oncogene protein	MYC
66	Prohibitin	PHB
67	5′-AMP-activated protein kinase catalytic subunit alpha-1	PRKAA1
68	Major prion protein	PRNP
69	Regulator of G-protein signaling 2	RGS2
70	60S ribosomal protein L13	RPL13
71	40S ribosomal protein S19	RPS19
72	40S ribosomal protein S26	RPS26
73	Plasminogen activator inhibitor 1 RNA-binding protein	SERBP1
74	Alpha-1-antitrypsin	SERPINA1
75	Sterol regulatory element-binding protein 2	SREBF2
76	Thymidylate synthase	TYMS
77	UL16-binding protein 2	ULBP2
78	Caspase-10	CASP10
79	Protein canopy homolog 2	CNPY2
80	Enoyl-CoA hydratase	ECHS1
81	60 kDa heat shock protein	HSPD1
82	Interleukin-32	IL32
83	Insulin receptor substrate 2	IRS2
84	Nucleophosmin	NPM1
85	Pyruvate dehydrogenase E1 component subunit alpha	PDHA1
86	Urokinase-type plasminogen activator	PLAU
87	Peptidyl-prolyl cis-trans isomerase A	PPIA
88	Retinoic acid receptor beta	RARB
89	60S ribosomal protein L18	RPL18
90	40S ribosomal protein S15	RPS15
91	Protransforming growth factor alpha	TGFA
92	UBX domain-containing protein 1	UBXN1
93	Zinc finger protein 593	ZNF593
94	Beta-2 adrenergic receptor	ADRB2

### Component-Target Network Analysis

**FIGURE 3:**
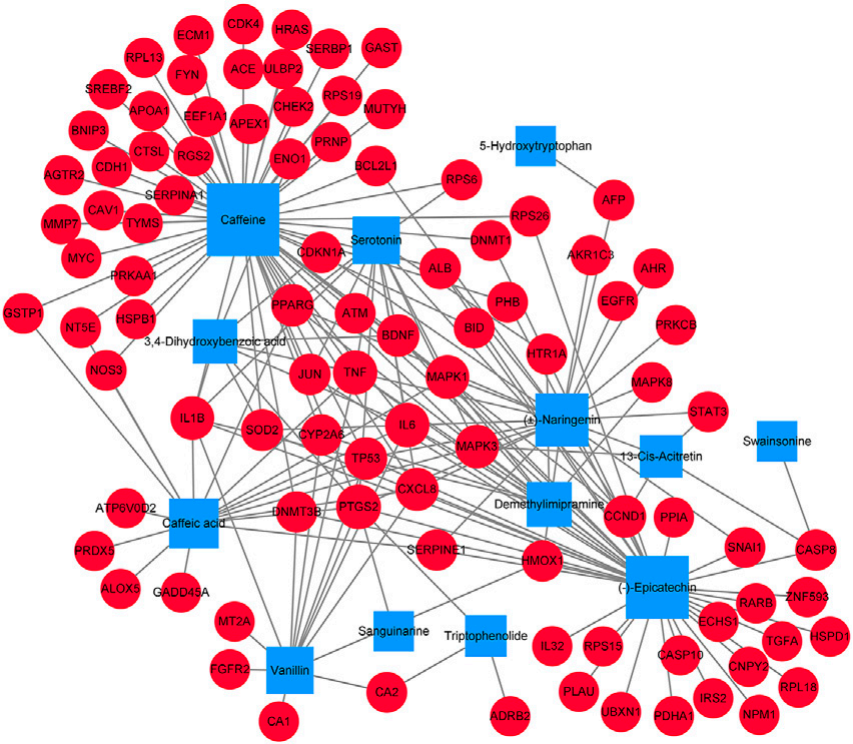
Component-target network of *D. officinale* extract and gastric cancer. The blue nodes represent candidate active components, and the red nodes represent potential protein targets. The edges represent the interactions between them, and the node sizes are proportional to the node degrees.

In order to better understand the potential mechanism of the *D. officinale* extract on gastric cancer, we used software Cytoscape 3.8.2 to construct the component–target network and component–target–pathway network. As shown in Figure 3, the component–target interaction network has 13 composite nodes and 94 target nodes with 191 edges. The network indicated the potential relationship between active substances and targets, thus revealing the potential mechanism of the *D. officinale extract* in the treatment of gastric cancer. The active substance with the highest degree of connection to the target is likely to be the substance that plays a major role in the treatment of gastric cancer. The active substance with the highest degree value was Caffeine (degree = 57), the active substances (−)-Epicatechin and (±)-Naringenin also had high degree values, which were 40 and 23, respectively. This indicates that a single compound can affect multiple targets, which may be related to the role of the *D. officinale* extract in the treatment of gastric cancer.

From the analysis of the target nodes, PTGS2, IL6, and TNF were associated with 9, 8, and 8 active substances, respectively. IL1B, MAPK1, and MAPK3 were associated with seven active substances, respectively; cXCL8 was associated with six active substances. These results showed that a variety of compounds could act on a single target in an interactive manner, indicating that they play a therapeutic role in gastric cancer through multiple components and multiple targets.

### PPI Network of *Dendrobium officinale* Extract-Related GC Target

**FIGURE 4:**
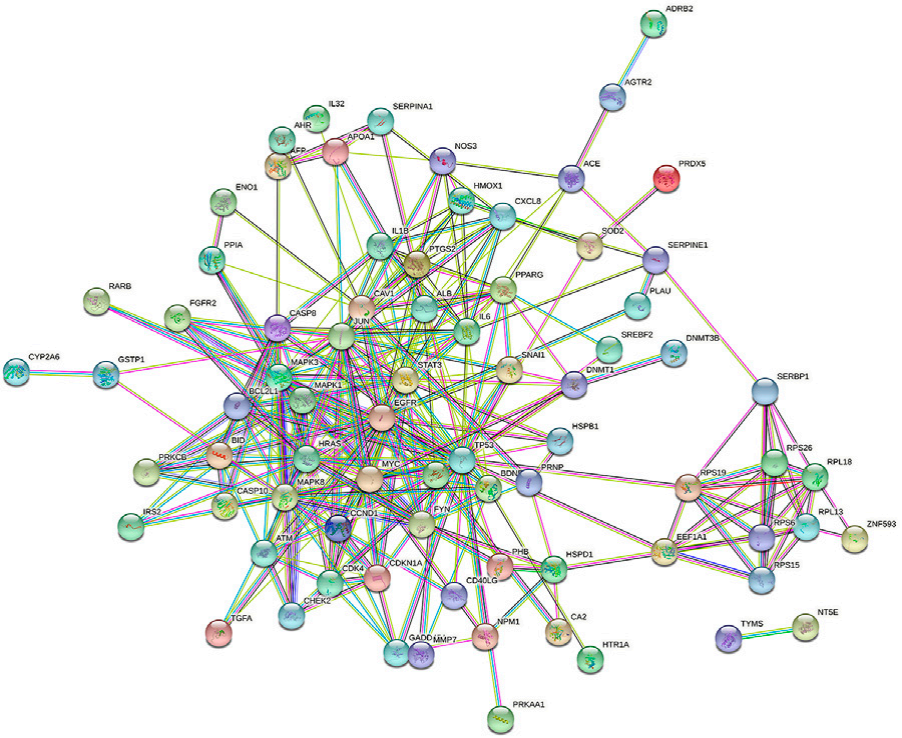
Protein–protein interaction (PPI) network of *D. officinale* extract-related targets in gastric cancer.

**FIGURE 5:**
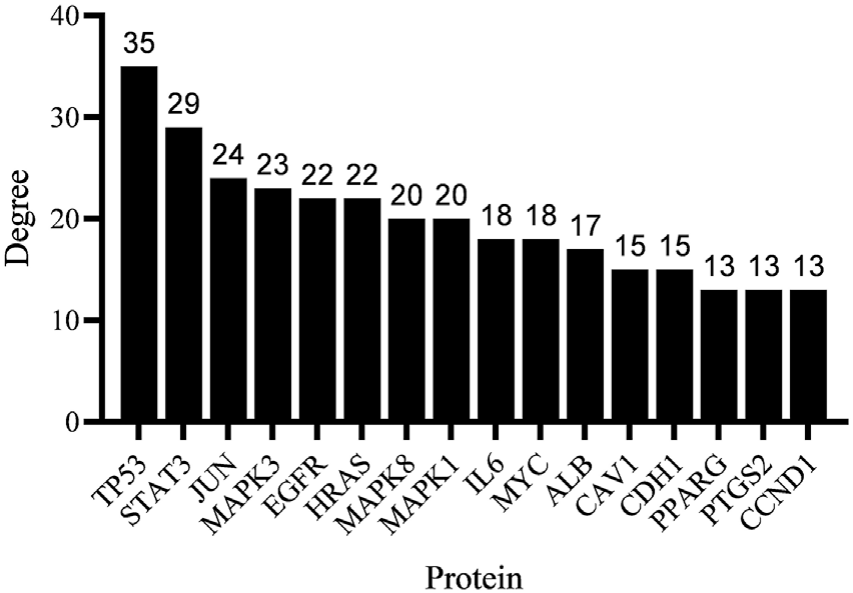
The degree value of the target protein (20%).

The PPI network was constructed based on the protein interaction screened from the related targets of the *D. officinale* extract on gastric cancer. The PPI network in Figure 4 contains 91 nodes and 135 boundaries. In the PPI network, nodes with larger degree values often play a more important role (Figure 5; Supplementay Table S3). It can be seen that the degree values of TP53, STAT3, JUN, MAPK3, EGFR, HRAS, MAPK8, MAPK1, and other eight target proteins are not less than 20, which are likely to be the key targets of the *D. officinale* extract for gastric cancer.

### GO and KEGG Pathway Enrichment Analysis

**FIGURE 6:**
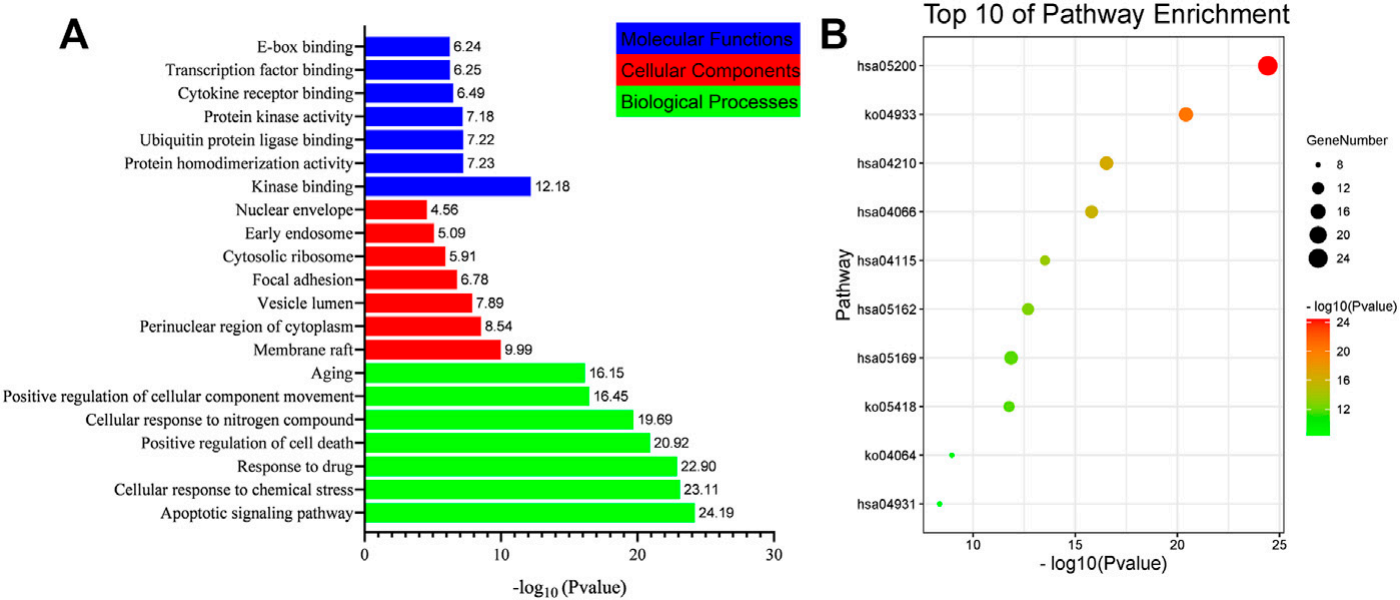
List of Gene Ontology (GO) and Kyoto Encyclopedia of Genes and Genomes (KEGG) pathway enrichment results in relation to the potential targets of *D. officinale* extract. **(A)** The first 21 GO terms were identified based on *p* < .01. **(B)** The top 10 pathways were identified based on *p* < .01.

In order to verify the biological characteristics of target genes related to gastric cancer treatment by the *D. officinale* extract, the GO enrichment analysis of the obtained proteins was performed using the Metascape database (*p* < .01, Figure 6A). The results showed that these proteins were related to 20 molecular functions, among which kinase binding, protein homodimerization activity, and protein kinase activity accounted for a higher proportion. Correlated with 16 cell types, the perinuclear region of the cytoplasm is the first. These proteins were related to 20 biological processes, with the largest association with apoptotic signaling pathway, positive regulation of cell death, cellular response to nitrogen compound, and response to drug (Supplementay Table S4 **)**.

**FIGURE 7:**
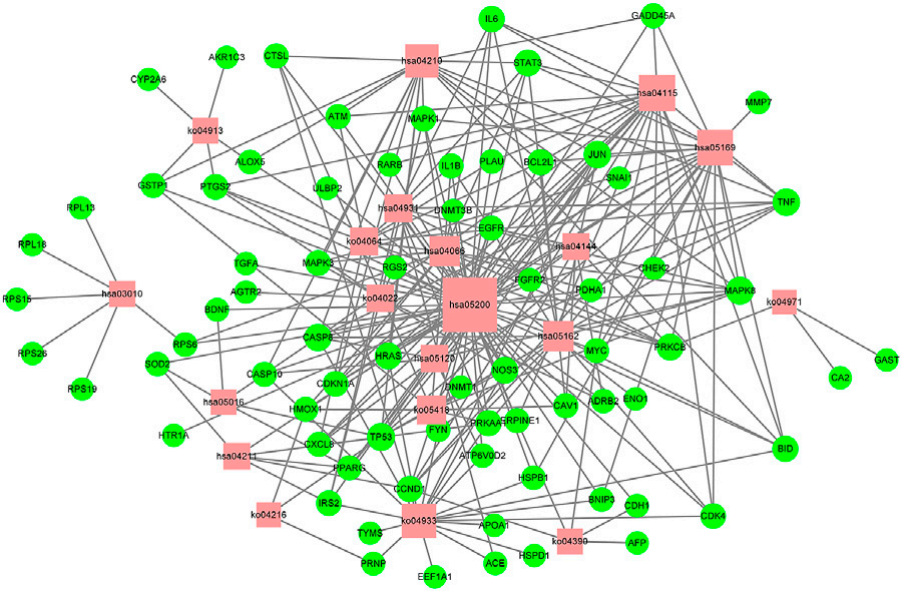
Target-pathway network of the *D. officinale* extract. The red nodes represent the pathways, and the green nodes represent the targets. The size of the nodes is proportional to the degree of the node.

In order to verify the correlation between the biological process involved in the target protein and the occurrence of gastric cancer, KEGG pathway analysis was performed for the target proteins (*p* < .01), and 20 signaling pathways were identified (Figure 6B). These signaling pathways included Pathway in cancer, the AGE-RAGE signaling pathway in diabetic complications, apoptosis, Epstein–Barr virus infection, HIF-1 signaling pathway, fluid shear stress and atherosclerosis, and the p53 signaling pathway (Supplementay Table S5). To better elucidate the relationship between target proteins and signaling pathways, we used Cytoscape 3.8.2 to construct a target-signaling pathway network (Figure 7) consisting of 94 nodes (20 signaling pathways and 74 target proteins) and 249 edges.

**FIGURE 8:**
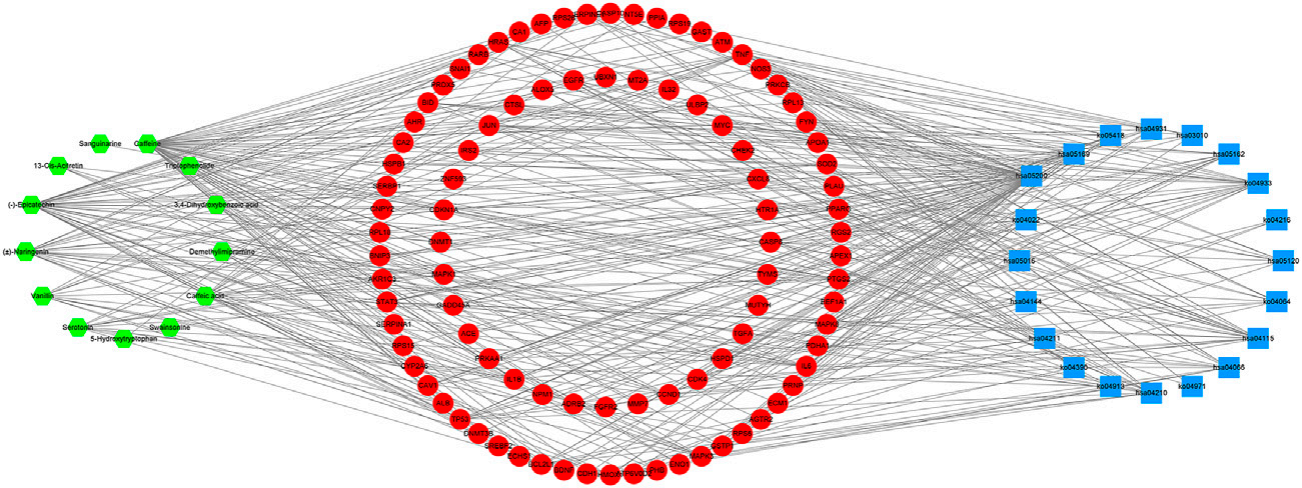
Component–target–pathway network of the *D. officinale* extract. The green nodes represent the component, and the red nodes represent the targets and the blue nodes represent the pathways.

In order to provide further illustration of the regulatory effect of active components of *D. officinale*’s extract on signaling pathways through target proteins, we constructed a component–target–signaling pathway network diagram (Figure 8), which included 127 nodes (including 13 active substances, 94 target proteins, and 20 signaling pathways) and 439 edges. Among the 94 target proteins, TNF, PTGS2, IL6, TP53, MAPK3, MAPK1, MAPK8, IL1B, JUN, and CASP8 were identified as relatively high-participation molecules, indicating that these molecules may play an important role in the development of GC.

### *Dendrobium officinale* Extract Inhibited GC Proliferation

**FIGURE 9:**
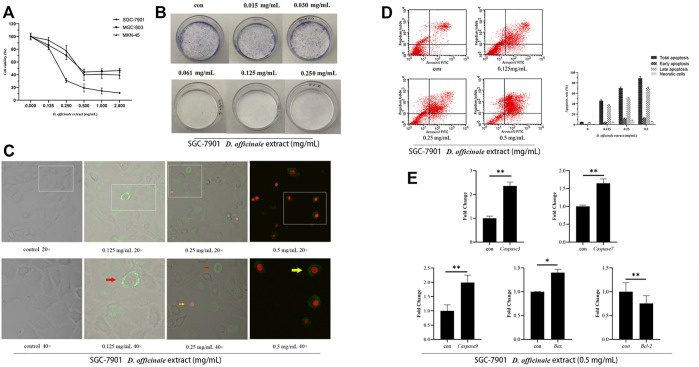
*D. officinale* extract exhibited anti-cancer effects in SGC-7901, MGC-803, and MKN-45 cells. **(A)** The cell viability was assessed by MTT assay after *D. officinale* extract administration for 24 h. The cells were incubated with 0–2 mg/ml of *D. officinale* extract. *n* = 3. **(B)** Colony formation in the presence of *D. officinale* extract. n = 3. **(C)** Confocal laser analysis of GC cells treated with *D. officinale* extract. *n* = 3. **(D)** Apoptosis analysis of *D. officinale* extract-treated GC. *n* = 3. **(E)** Fluorescence quantitative detection of *Caspase-3*, *Caspase-7*, *Caspase-9*, *Bax*, and *Bcl-2* after treating GC cells with .5 mg/ml *D. officinale* extract for 4 h *p < .05, **p < .01 vs. con.

In order to verify the effect of the *D. officinale* extract on the proliferation of gastric cancer cells, a series of cytological experiments was performed. The MTT assay was conducted to identify the effects of the *D. officinale* extract on the viability of SGC-7901, MGC-803, and MKN-45 cells. After exposure to 0–2 mg/ml of the *D. officinale* extract for 24 h, a dose-dependent decrease in cell viability was observed (Figure 9A). The IC_50_ (half-maximal inhibitory concentration) values of the *D. officinale* extract in SGC-7901, MGC-803 and MKN-45 cells were 0.160, 0.250 and 0.285 mg/ml, respectively.

Here, we examined the anticancer effect of the *D*. *officinale* extract in SGC-7901 cells. The cells were exposed to the *D. officinale* extract (.015–.250 mg/ml) for 24 h. The colony formation assay was used to detect the ability of SGC-7901 cells to form colonies in the presence of the *D. officinale* extract. The results showed that the *D. officinale* extract could significantly reduce the formation of SGC-7901 cell colonies (Figure 9B). In addition, the results of laser confocal microscopy and flow cytometry showed that the *D. officinale* extract could induce SGC-7901 cell apoptosis (Figures 9C,D).

## Discussion

We explored the molecular mechanism through which the *D. officinale* extract inhibited the proliferation of gastric cancer cells by network pharmacology. In this paper, a series of databases were used to screen the potential active substances of the *D. officinale* extract and identify their related targets. The protein interaction network identified the key proteins associated with the inhibition of gastric cancer proliferation by the *D. officinale* extract. The results showed that the *D. officinale* extract directly or indirectly interacted with eight key targets such as TP53, STAT3, JUN, MAPK3, EGFR, HRAS, MAPK8, and MAPK1, thus exerting anticancer effects.

TP53 connects 35 nodes in the PPI network. Its main functions are to control cell-cycle progression, cell senescence, DNA repair, apoptosis, and the inhibition of angiogenesis. The inhibition of TP53 promotes the development of gastric cancer (Feng et al., 2019), which means that TP53 acts as a tumor suppressor in human gastric cancer. EGFR is a transmembrane glycoprotein that connects 22 nodes in the PPI network. The activation of EGFR may lead to the activation of multiple signaling pathways, thereby regulating cell proliferation and differentiation (Komposch and Sibilia, 2015). MAPK8, MAPK1, and MAPK3 are members of the MAPK family, and they are involved in cell proliferation, differentiation, migration, transformation, and apoptosis. JUN, MAPK8, MAPK1 and MAPK3 are signal proteins in the MAPK signaling pathway, and they are involved in the process of apoptosis (Li et al., 2021). Therefore, these proteins are the key targets of the *D. officinale* extract inhibiting gastric cancer cell proliferation.

The main feature of cancerous cells is that they are immortalized without apoptosis. Screening assays for antitumor compounds in natural plants have revealed that the target substances have cytotoxic effects, that can directly kill cancer cells rather than through the induction of apoptosis (Hengartner, 2000). Screening for compounds that can induce apoptosis in tumor cells is conducive to reducing the toxicity of drugs and lead to better development prospect. In this paper, GO enrichment analysis and KEGG pathway analysis were performed, and it was found that the potential active components of the *D. officinale* extract could act on signaling pathways such as axon guidance and the MAPK signaling pathway. The activation of the caspase signal transduction pathway can play an anticancer role by inducing SGC-7901 cell apoptosis.

Members of the caspase family of proteins play an important role in the regulation of apoptosis (McIlwain et al., 2013; Wnek et al., 2017; Van Opdenbosch and Lamkanfi, 2019). *Bcl-2* family members are important regulators of the endogenous pathway of mitochondrial initiation. Based on their regulatory function, proteins in the *Bcl-2* family are divided into two categories, antiapoptotic and apoptotic. The anti-apoptotic members include *Bcl-2*, *Bcl-xl*, and *Mcl-1* and apoptotic members include *Bak* and *Bax*. *Bcl-2* family members form dimers, which interact with each other, affecting the permeability of mitochondrial membrane, releasing apoptosis-related factors and regulating apoptosis (Nagata, 2018). Quantitative fluorescence experiments showed that the expression levels of *Caspase-3*, *Caspase-7*, *Caspase-9*, *Bax*, and other genes in SGC-7901 cells treated with the *D. officinale* extract at different times were significantly upregulated, while the expression level of *Bcl-2* decreased (Figure 9E), this was consistent with the results of the network pharmacology analysis.

## Conclusion

In this study, 13 potential active components were identified by screening the extract of *D. officinale*. Through a network pharmacology analysis, it was clarified that the *D. officinale* extract inhibited the proliferation of gastric cancer cells through various signalling pathways in cancer, including PI3K signaling pathway, WNT signaling pathway, and HIF-1 signaling pathway. The expression of *Bcl-2* was inhibited and the expression of *Bax* was upregulated. Moreover, the expression levels of *Caspase-3*, *Caspase-7*, and *Caspase-9* were upregulated, and the activation of the caspase signaling pathway led to the development of apoptosis.

## Data Availability

The original contributions presented in the study are included in the article/Supplementary Material; further inquiries can be directed to the corresponding author.
